# Designing and Implementing a Home-Based Couple Management Guide for Couples Where One Partner has Dementia (DemPower): Protocol for a Nonrandomized Feasibility Trial

**DOI:** 10.2196/resprot.9087

**Published:** 2018-08-10

**Authors:** Reena Lasrado, Therése Bielsten, Mark Hann, Linda Davies, James Schumm, Siobhan Reilly, Caroline Swarbrick, John Keady, Ingrid Hellström

**Affiliations:** ^1^ Division of Nursing, Midwifery & Social Work The University of Manchester Manchester United Kingdom; ^2^ Department of Social and Welfare Studies Linköping University Norrköping Sweden; ^3^ Division of Population Health, Health Services Research & Primary Care The University of Manchester Manchester United Kingdom; ^4^ IT Services The University of Manchester Manchester United Kingdom; ^5^ Division of Health Research Lancaster University Lancaster United Kingdom; ^6^ Department of Health Care Science Ersta Sköndal Bräcke University College Stockholm Sweden

**Keywords:** dementia, couple management guide, dementia self-help, dementia intervention

## Abstract

**Background:**

The increasing rate of dementia and high health and social care costs call for effective measures to improve public health and enhance the wellbeing of people living with dementia and their relational networks. Most postdiagnostic services focus on the condition and the person with dementia with limited attention to the caring spouse or partner. The key focus of the study is to develop a guide for couples where one partner has a diagnosis of dementia. This couple management guide is delivered in the form of an app, DemPower.

**Objective:**

This study aims to investigate the feasibility and acceptability of DemPower and to assess the criteria for a full-integrated clinical and economic randomized control trial. DemPower couple management app will be introduced to couples wherein one partner has dementia.

**Methods:**

The study will recruit 25 couples in the United Kingdom and 25 couples in Sweden. Couples will be given 3 months to engage with the app, and the amount of time taken to complete the guide (can be <3 or >3 months) will be reviewed. A set of outcome measures will be obtained at baseline and postintervention stages.

**Results:**

The proposed study is at the recruitment phase. The DemPower app is being introduced to couples from consultation groups at a pretrial phase for identifying any bugs and exploring if any navigation challenges exist. The feasibility testing will begin in April 2018.

**Conclusions:**

The study will determine how much support couples need to engage with DemPower and whether or not they make use of it in their everyday lives. If there is support for app use, a future study will assess whether it is superior to “usual care.”

**Trial Registration:**

International Standard Randomized Controlled Trial Number (ISRCTN): 10122979; http://www.isrctn.com/ISRCTN10122979 (Archived by WebCite at http://www.webcitation.org/70rB1iWYI)

**Registered Report Identifier:**

RR1-10.2196/9087

## Introduction

The prevalence of dementia is rising globally with increases in the average age of the world’s population and is estimated to reach 131.5 million by 2050 [1]. The evidence base for high-income countries (United States, United Kingdom, Netherlands, and Sweden) suggests a declining trend in age-specific incidence [2,3]. In the United Kingdom and Sweden, two-thirds of all people with dementia are aged over 80 years [4,5] and more than 50% live alone at home or with their care partner [6,7]. In 2013, the total health and social care cost of dementia in the United Kingdom was estimated to be £26.3 billion, of which 44% were unpaid care costs [5,8]. In Sweden, during 2012, the estimate for health and social care costs was SEK 52 billion, of which informal care was 16.7% [2]. The financial costs to health and social care that are attributed to dementia are likely to continue to increase in future years unless effective measures are undertaken to improve public health [4,9]. Accordingly, a well-defined approach needs to be adopted to manage the implications of dementia on societal systems and organizations, including the wellbeing of people living with dementia and their relational networks [1].

The national and international push for early diagnosis and intervention in dementia has incentivized programs and strategies to improve diagnostic rates and quality of life for people living with dementia and their families. In the United Kingdom, some of these strategies involve public awareness campaigns, training health and social care professionals, memory assessment and support services, internet-based forums for caregivers, and creating dementia-friendly infrastructures, spaces and technology [10-15]. However, despite these improvements, some studies indicate that people with a recent diagnosis of dementia are largely left on their own to manage their condition [16,17] and are only (re)visited by statutory services once a crisis at home occurs [7]. This is an interesting paradox as the demographic data suggest that most people with dementia want to live in their own home and neighborhood for as long as possible. It is, therefore, imperative that relevant supports are made available early on during the diagnostic process, and that people living with dementia and their relational networks are made aware of available resources and services. This could prevent the occurrence of crisis situations and reduce health and social care costs.

In the United Kingdom, alongside mainstream health and social care services, some charities such as the Mental Health Foundation, Age UK and the Alzheimer’s society have long provided localized support services and opportunities for people with dementia and their families to access community engagement programs. While welcome, there remains a dearth of dyadic, couple-centered approaches [18,19], with most interventions for couples where one partner has a dementia overwhelmingly emphasizing the condition (the dementia), rather than on the needs of partners as a couple [20-24]. In Sweden, most people with dementia live in their homes, in which they require care and support. This has been identified as one of the main challenges facing primary care in Sweden [25]. To address this gap in interventions for couples, an easy to use couple management guide was developed as part of the “Living Life and Doing Things Together” project. This project is one of eight work programs within the Economic and Social Research Council (ESRC)/National Institute for Health Research (NIHR) Neighbourhoods and Dementia mixed methods study [26]. All the eight work programs are underpinned by a neighborhoods’ model [27], positioning people living with dementia and their social and relational networks at the center of the overall study aims and objectives. Informed by this approach, the current work program (6) is centered on the lives of couples who live together at home, where one partner has a diagnosis of dementia. The couple management guide developed in this study is delivered in the form of an app, DemPower*.* In this study, the intervention was tested for feasibility.

The use of mobile apps in health care, especially among people with dementia and their families, has gained prominence due to the ability of these apps to promote quality of life for the person with dementia and their families [28,29]. Some of the apps on the market include global positioning system trackers to enable people with dementia and their families to feel safe in their neighborhoods [30,31], schedule activities of daily living, communicate, and manage appointments and emergency help [32]. The wider research evidence suggests self-management resources in the form of apps, complemented with appropriate training and support, are beneficial for patients in the early to moderate stages of dementia and enhance quality of life [28,33]. This paper reports the protocol to evaluate the acceptability and feasibility of the DemPower intervention.

The overall aim of the study is to investigate the feasibility and acceptability of the app-based couple management guide DemPower among couples living together at home, where one partner has dementia. The key objectives are (1) to explore if the DemPower couple management guide is useful and acceptable to couples, (2) to assess the recruitment capability and how effective and appropriate are the outcome measures and data collection procedures, and (3) to analyze the potential for conducting an economic evaluation in the full trial.

## Methods

### Study Design

The study uses a prospective, nonrandomized feasibility design to investigate the level of uptake of DemPower among couples where one partner has a diagnosis of dementia. The methodology facilitates an assessment of study processes to explore the criteria for a full trial.

### Study Population

The study will involve couples with one partner having a diagnosis of dementia and who live together at home. We will seek to recruit a wide and diverse couple population. This extends to any type of dementia, sexual orientation, age, profession, social, cultural, and religious contexts. Participants will be enrolled regardless of comorbidities experienced by a partner with dementia and the health status of a supporting partner. We will also include participants enrolled in other research studies. The wide diversity of participants is envisaged to study a better understanding of potential participants for a future full trial. This also provides opportunities to explore population needs, compare outcomes, and inform the areas that need further development. Specific inclusion and exclusion criteria are listed in Textbox 1.

### Sample Size

**Figure 1:**
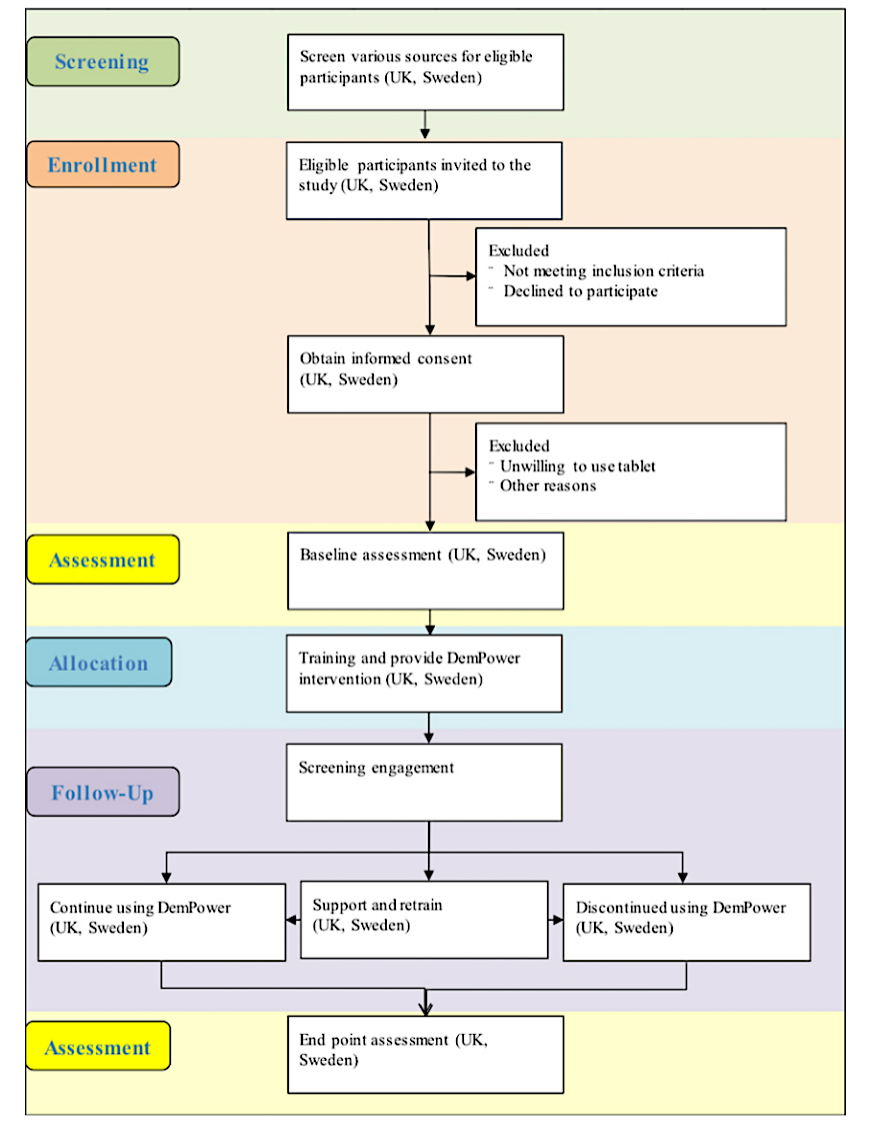
Study stages.

As this is a feasibility study, no formal power calculation is needed. Instead, Lancaster et al [34] recommend including between 30 and 50 participants (in this study, couples) to estimate critical parameters (eg, recruitment rate; standard deviation of primary outcome) with necessary precision. This study does not aim to randomize participants, and therefore, there is no control group in this study. We will aim to recruit 50 couples across North West England and Sweden (Linköping and Norrköping). Figure 1 describes various stages of the study.

### Selection and Recruitment

#### In the United Kingdom

The recruitment of 25 couples in North West England will take place via Clinical Research Network, Joint Dementia Research network, dementia cafes, and third sector organizations. We will also advertise our study in memory clinics and through the wider project’s website and social media (Twitter) networks to identify and recruit potential participants who are interested in the study.

Staff members working at National Health Service Trusts and Clinical Research Networks will be involved in identifying people with dementia to the study, and they will use the Trust databases and the Join Dementia Register to identify potential eligible participants.

The researcher will establish contacts with staff from third sector organizations and coordinators of dementia cafes (eg, Age UK, Alzheimer’s society, Creative Mind etc). Appropriate permissions will be sought prior to addressing the group. The study poster will be displayed at these meetings, and detailed study information will be available for interested group members. The researcher will ensure that the third sector organizations’ contact source or coordinators do not communicate potential participants’ details without their prior permission.

For recruiting through advertisements, interested participants are free to contact the researcher. Contacts established in this manner will be followed up through a face-to-face meeting. At this meeting, the researcher will explain the study and hand in the detailed study information pack.

Potential participants who meet the inclusion criteria will be given information about the study and the opportunity to express an interest in taking part in the study. Potential participants will have opportunities to ask any questions they have and to discuss any aspects of the study with the researcher before they make their decision. The interested participants will be given sufficient time to make a decision and be approached again to obtain informed consent (24 hours or more after the first visit).

#### In Sweden

A total of 25 couples will be recruited from memory clinics and dementia cafes based in county Östergötland (Linköping and Norrköping) of Sweden.

The staff at the memory clinic will identify couples, contact potential participants to discuss the study, and identify couples who are interested in participating. Couples who express interest in participating will have their details passed on to the relevant researcher.

The researcher will contact study staff or coordinators within the third sector organizations (eg, dementia cafes): day care centers and family care centers within the municipalities of Linköping and Norrköping. On obtaining relevant permissions, study posters will be displayed and details of the study will be presented to the members of the group. Researchers will approach interested participants individually to assess for inclusion, discuss the study in detail, and obtain consent.

**Inclusion and exclusion criteria.**

**Inclusion criteria**

- Couples who have a partner or spouse with a diagnosis of dementia in early to moderate stages. The stage will be identified either via clinical team during referral or through self-report
- Couples who live together in their own homes (not residential care).
- Both partners understand and speak English (in the United Kingdom) or Swedish (in Sweden)
- Couples in a long-term relationship for 2 or more years

**Exclusion criteria**

- Couples with one or both partners who are blind and might find it difficult to interact with DemPower
- Any partner who has become completely immobile or bed-bound and may not be able to engage with suggested activities
- Both partners having a diagnosis of dementia
- Couples where one or both partners lack capacity or may have fluctuating capacity

### DemPower Intervention

Living Life and Doing Things Together is a couple management guide delivered through the platform of an electronic application on a touchscreen device (Android tablet). The guide is aimed at enhancing the wellbeing as well as the relationship of couples where one partner has a diagnosis of dementia. The contents in this guide are based on couples with lived experience, available research, and practice evidence.

The objectives of the DemPower guide are (1) to help couples focus on the activities that they can do rather than what they cannot, (2) to reflect upon the strengths of their relationship, (3) help couple find ways to tackle daily activities together, and (4) to archive reflections, moments, and memories the way they would like them to be remembered.

**Figure 2:**
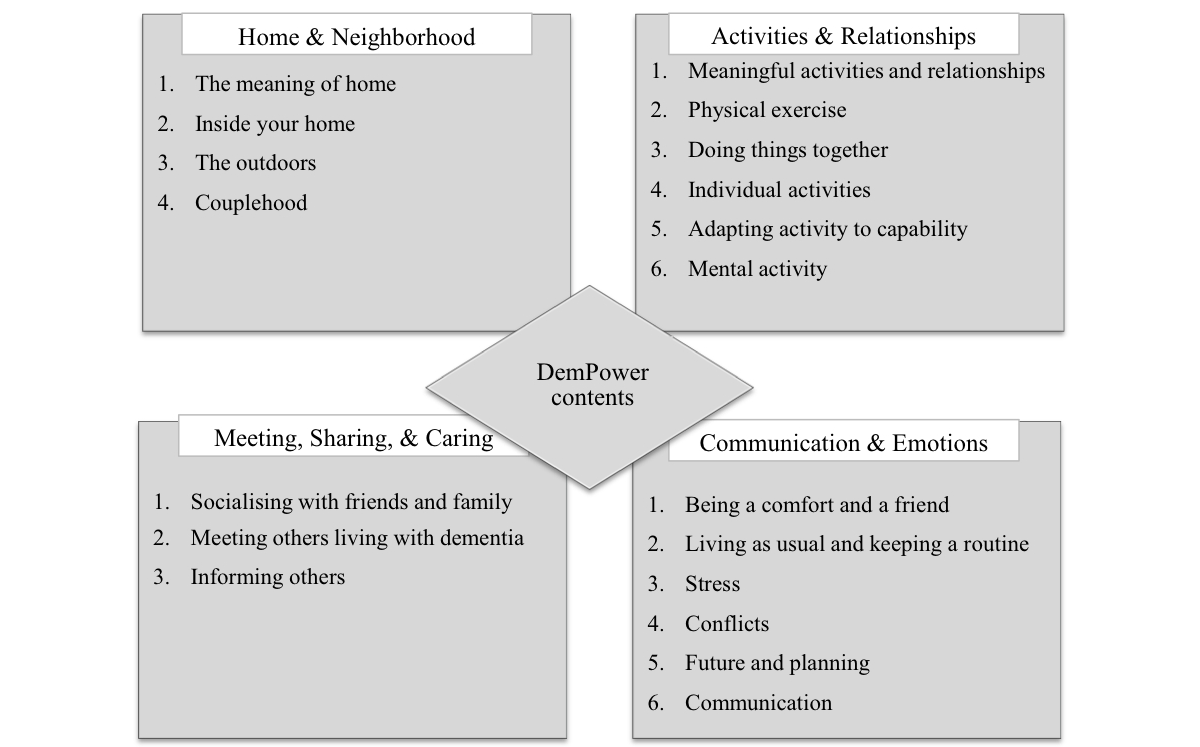
Guide contents.

The guide is structured under 4 primary themes and several sections within each theme, as illustrated in Figure 2. The themes and corresponding sections are introduced using storyboard techniques with a voiceover. Videos clips of couples sharing their experiences demonstrating particular situations follow storyboard illustrations. The guide makes suggestions for activities under each section. Examples of some suggested activities include games (stored on the device), links to useful information, taking pictures, writing reflections, and discussing emotions, needs and required changes to their home and their approach to daily life.

The app design facilitates various forms of user interfaces and provides users the control over font size, color, language, volume etc. The design also focuses on making the interface simple and easy to access. The interaction within the app is multimodal (voice, touch, text to speech). User-centered and participatory approaches inform the overall app design and concept.

The app and all the corresponding resources will be saved onto a portable device (tablet) prior to handing them over to the recruited couples (one tablet per couple). Researchers will provide a tutorial on how to use the device and the app. A detailed help video is inbuilt into the app. Researchers will provide a printed copy on how to use the device and the guide to all the participants. The participants may contact researchers at any time if they require technical support. The researchers will call the participants once a month to check their progress and arrange visits if required. We will recommend the participants to engage with two sections per week. They have the option to engage with a selection of sections or with the entire guide and in any order. Participants are expected to complete the guide within a period of 3 months from the date of device activation.

### Feasibility, Acceptability, and Health Outcome Measures

The goal of the study is to explore feasibility, acceptability, and usability of DemPower among couples where one partner has dementia. Using Bowen et al’s [35] suggested areas of focus for feasibility studies, Table 1 reports the approaches and measures used to assess the different aspects of the feasibility and acceptability of the DemPower intervention [35-37]. The structure in Table 1 helped to identify relevant outcomes, demonstrate how these address study objectives, and inform the choices of data collection procedures.

Two primary data sets will be generated from the study: evaluation and outcomes data. The study team designed a set of evaluation questionnaires to assess participants’ experiences with DemPower and the study processes (Multimedia Appendices 1-5). These questionnaires, DemPower, and chosen outcome measures will be tested with couples from a consultancy group for comprehension errors and bug fixing prior to the feasibility phase. The outcomes used to represent effectiveness include health-related aspects of quality of life, self-efficacy, interconnectedness, and mutuality on the part of both partners.

**Table 1:** Bowen et al’s [35] feasibility criteria and study application.

Area of focus	Study application	Outcomes of interest	Data sources
Acceptability and integration	To what extent is DemPower suitable to implement in home-living couples with dementia?	Perceived acceptability and satisfaction	Evaluation Questionnaires, 3-point Likert scale
Demand	To what extent do couples consider the couple management approach and the content of DemPower as desirable support?	Perceived usability	Usage tracking data, self-reported system usability scale [38], 5-point Likert scale
		Perceived benefits of DemPower	Evaluation Questionnaires, 3-point Likert scale
Implementation and practicality	To what extent can DemPower be successfully implemented with couples living with dementia?	Willing and able to complete all the sections of the self-help guide	Dropout rates usage tracking data
		Type of, and amount of support needed by couples	Evaluation questionnaire – custom matrix
		Degree of technical errors, resources, factors influencing implementation	Researcher log
Adaption	To what extent can DemPower be used in its current state?	Degree of errors; levels of support	Researcher log; Evaluation Questionnaires, 3-point Likert scale
Efficacy	To what extent does DemPower show promise of encouraging engagement and positive effects on couples’ relationships and beliefs in managing daily life?	Impact on quality of life, relationship quality, mutuality, closeness, sense of couplehood, self-efficacy, and health status	Baseline and post intervention or end point assessments of quality of life—Quality of Life in Alzheimer´s disease [39], Care-related Quality of Life [40]; Relationship Quality and Mutuality – Mutuality Scale [41]; Closeness and interconnection – Inclusion of Other in Self Scale [42]; Empowerment /Self-efficacy – General Self-efficacy Scale [43]; Health status – EQ-5D-5L (EuroQol health measure with 5 levels of severity for 5 dimensions) [44]

Table 2 presents the measures used to assess the potential effectiveness of the DemPower app and provide information for the design of any future randomized controlled trial (RCT), including sample size estimation.

### Data Collection

An evaluation questionnaire will explore participants’ experiences with the guide, relevance of guide’s contents, usability of the app, perceived benefits, relevance of the guide in everyday living, applicability to various contexts, the volume of contents, and the time required to complete the guide (Multimedia Appendix 1). Responses to questions may vary from rating, multiple choices to descriptive forms.

At the end of each section, written instructions will be provided, guiding participants to use a particular set of questionnaires. Questionnaires are split across 4 themes presented in the guide (Multimedia Appendices 2-5). Researchers will follow up on participants’ progress with the questionnaires during regular telephonic conversations or home visits and offer support if required.

The following usage data will be stored on the tablet to measure how the app is used:

- The number of times and timestamp of when each screen was viewed
- The number of times the initial splash or landing screen has opened, in order to tell how many times app was started from scratch
- The contents pages will indicate how users are navigating the app, whether by the contents pages or going through sequentially using next or back buttons.

The outcome measures, questionnaires, and usage tracking data will form primary sources of data for this part of the study. All the questionnaires will be administered using an offline or a paper-based survey tool. The researcher will administer the outcome measures listed in Table 2 at baseline (prior to introducing the guide) and at the end point. Participants who choose to engage with only parts of the intervention will also be assessed at the start and at the time that they declare as completed or at the end of 3 months from the intervention initiation date. The details of participants’ progress, telephonic follow-ups, home visits, and variations in follow-up time frames will be recorded in the researchers’ logs. Researchers will support the person with dementia as he or she completes the outcome measures at baseline and at the end point during face-to-face meetings. Spouse or partner caregivers will be encouraged to complete the assessments on their own during the researcher’s visit.

### Feasibility Design Process

The outcome relevant to the design process is to ascertain if a main study is feasible by classifying the study under one of the following categories: (1) Stop – main study not feasible; (2) Continue, but modify protocol – feasible with modifications; (3) Continue without modifications, but monitor closely – feasible with close monitoring, and (4) Continue without modifications – feasible as is.

The evaluation of the study process will be informed by Medical Research Council’s guidance on complex interventions [36], and principles and questions specific to feasibility designs discussed by Bowen et al [35] and Osmond and Cohen [46].

**Table 2:** Outcomes of effectiveness.

Outcomes and tools		Description	Answered by
**Quality of Life**			
	Quality of life in Alzheimer´s disease [39]	13-item tool Addresses mood, cognitive, and functional ability, activities of daily life and quality of relationships with family and friends Uses a 4-point Likert scale ranging from “poor” (1p) to “excellent” (4p) with a maximum score of 52	Both the spouses or partners individually
**Caregiver-Related Quality of Life**			
	Carer Quality of life [40]	7-item tool Addresses 5 negative and 2 positive dimensions of providing informal care Uses 3-point Likert scale from “a lot” (0p) to “no” (2p) for the negative dimensions and reversed scale for positive dimensions. The higher the score, the better the care situation	Partner or spouse caregiver
**Self-efficacy**			
	General Self-efficacy Scale [43]	10-item tool Assess coping skills and adaptation to situations Has a four choice response ranging from “not at all true” (1p) to “exactly true” (4p); Scores are summarized to a total score, and a higher score indicates a higher sense of self-efficacy	Both the spouses or partners individually
**Interconnectedness**			
	The Inclusion of Other in Self Scale [42]	A single item pictorial measure of closeness Assess people’s sense of being interconnected to each other	Both the spouses or partners individually
**Mutuality**			
	Mutuality Scale [41]	15-item Mutuality Scale Includes four dimensions—love and affection, shared values, reciprocity and shared pleasurable activities Rated on a 4-point Likert scale between 0 “not at all” to 4 “a great deal”	Both the spouses or partners individually
**Health and social care service use**			
	Service use questionnaire	The service use questionnaire was adapted from current service use questionnaires held by the investigators, it will be refined by consultation with the study service user group Covers key health and social care services Assesses the range of services used as well as the frequency of use The measure will be administered by the researcher at the baseline and end of follow-up assessments	Both the spouses or partners individually
**Health status**			
	EQ-5D-5L (EuroQol health measure with 5 levels of severity for 5 dimensions) [44]	Has a 5-dimensional structure (mobility, self-care, usual activities, pain or discomfort, and anxiety or depression) Each dimension has 5 levels: no problems, slight problems, moderate problems, severe problems, and extreme problems Allows estimation of quality-adjusted life years	Both the spouses or partners individually
	DEMQOL (Measurement of health-related quality of life for people with dementia) [45]	A condition-specific measure of health-related quality of life for people with dementia Can be completed with the person with dementia or a main caregiver The measures cover 5 domains: daily activities and looking after yourself, health and wellbeing, cognitive functioning, social relationships, and self-concept Preference weights are available to allow estimation of quality-adjusted life years	Partner or spouse with dementia

The process evaluation plays a key role in assessing the quality of study implementation, recruitment capability, attrition rate, data collection procedures, relevance of outcome measures and evaluation questionnaire, resource capability for implementing the study and acceptability of randomization. The study design does not allow for evaluating the randomization process; therefore, the acceptability of randomization will be explored with participants in the form of open-ended questions administered at the end of the study.

The data relevant to these areas will be obtained from researchers’ field notes, process evaluation questionnaires, recruitment and attrition data, contextual data associated with variations in outcomes, evaluating research team’s capabilities, researchers’ observations of suitability of outcomes, questionnaires, the burden on participants, and support required and will use results from the intervention segment to assess the relevance of chosen methodology.

### Data Analysis

All the data obtained from outcome measures and evaluation questionnaires will be entered into an Excel or Stata database for further statistical analysis. The data will be anonymized prior to being uploaded to a database. The qualitative or open-ended responses from evaluation questionnaires will be categorized and analyzed thematically. The data from Sweden will be transferred to the research team at the University of Manchester for statistical analysis.

Given that this is a feasibility study (and that there is no control group), we will not be carrying out hypothesis testing to determine if the intervention is effective. Instead, we will focus on (1) estimating recruitment and attrition rates; (2) determining whether there is sufficient change in potential outcome measures following the intervention, using appropriate descriptive measures of central tendency (mean, median) and spread (standard deviation, inter-quartile range, range); (3) estimating the standard deviation of potential outcome measures to inform or refine a sample size calculation for the Phase III trial; and (4) estimating response rates to participant questionnaires, checking for floor and ceiling effects and in fact whether the intervention is acceptable to participating couples. We will reconsider the use of questionnaires or other items if the amount of missing data exceeds 10%.

### Economic Evaluation

This study will not include a formal economic evaluation. Rather, the data collected will be used to inform the design and implementation of a full RCT to assess the (cost) effectiveness of the intervention. We will use the service data to identify the range of services used by participants. The service use data will be costed using published unit costs to estimate the likely health and social care costs. Descriptive statistics and regression analysis will be used to identify key cost drivers. Published utility weights will be applied to the EuroQol health measure with 5 levels of severity for 5 dimensions and the Measurement of health-related quality of life for people with dementia and used to estimate quality-adjusted life years. Descriptive statistics and regression analysis will be used to explore the level of association between the two measures and key domain drivers of overall utility and quality-adjusted life years.

### Ethical Considerations

The study is approved by the National Health Service Research Ethics Committee (17/NW/0431) in the United Kingdom and the Regional Ethical Review Board in Sweden (Dnr: 2017 2017/281-31).

The approach to obtaining consent is informed by a “process consent” method [47], whereby a researcher enables participants to make informed decision from the point of initial contact to completion of the study [48]. Guided by the Mental Capacity Act [49] and the process consent approach, the researchers will make every effort to ensure that the participants are provided with all the relevant information that they understand the information and are able to retain the information long enough to make a decision and are willing to participate in the study.

People who have capacity to consent will provide either a written or verbal consent. Participants who find reading or writing challenging will have an option of expressing consent verbally, and it will be audio recorded.

## Results

This is an on-going study, currently at the trial phase. The study is currently recruiting participants from both sites. This nonrandomized feasibility study will produce results pertaining recruitment capability, usability, and acceptability of DemPower among couples where one partner has dementia as well as the relevance of chosen outcome measures and evaluation questionnaires. The results will inform a future RCT and fully powered health economic assessment.

## Discussion

### Principal Findings

A scoping review, conducted earlier on in the study, highlighted the dearth of couple-oriented interventions for couples affected by dementia. Postdiagnostic dementia services and service uptake by people with dementia and their caregivers are quite diverse, depending upon population groups and geographical locations [2,9,50]. The DemPower guide attempts to combine the resources available and act as a single source of information, while providing strategies for managing everyday living. DemPower is particularly aimed at couples who live together at home and where one partner has a diagnosis of dementia. The contents of DemPower have been informed by the experiences of couples who live with dementia and daily life and social situations that include potentially important ways of maintaining a healthy relationship.

### Future Work

Through this feasibility study, we will be able to establish participants’ interest and engagement with the app, how useful and helpful participants find the sections on “home and neighborhood,” “activities and relationships,” and “approach and empowerment.” We also have the opportunity to explore how this app translates into everyday life. This information is crucial for exploring how usual care compares with DemPower and sets stage for a full RCT.

In terms of the study methodology, relevant information on recruitment and attrition rate will inform the future power calculation for a full trial. In the current feasibility study, we test the app in two countries (United Kingdom and Sweden); this will provide insights into if any systemic differences influence the uptake of the app. These data will inform the transferability of the app to various contexts, cultures, and countries.

### Limitations

The diverse cultural and social contexts in the United Kingdom and Sweden might raise some challenges for comparing data and estimating parameters for a full trial, although it does provide opportunities for identifying key areas for consideration in future multinational trials. The design of DemPower is limited and might require further adaptations to make it applicable to people with visual impairments. Finally, differences in recruitment strategies in the United Kingdom and Sweden might influence the recruitment and retention rate.

## Abbreviations

DEMQOL: Measurement of health-related quality of life for people with dementia
ESRC: Economic and Social Research Council
EQ-5D-5L: EuroQol health measure with 5 levels of severity for 5 dimensions
NIHR: National Institute for Health Research
RCT: randomized controlled trial

## Appendix

Multimedia Appendix 1 Process evaluation questionnaire.

Multimedia Appendix 2 App-specific feasibility questionnaire 1.

Multimedia Appendix 3 App-specific feasibility questionnaire 2.

Multimedia Appendix 4 App-specific feasibility questionnaire 3.

Multimedia Appendix 5 App-specific feasibility questionnaire 4.

## References

[1] Prince M, Comas-Herrera A, Knapp M, Guerchet M, Karagiannidou M (2016). World Alzheimer Report.

[2] Wimo A, Jönsson L, Fratiglioni L, Sandman P, Gustavsson A, Sköldunger A, Johansson L (2016). The societal costs of dementia in Sweden 2012–relevance and methodological challenges in valuing informal care. Alzheimer's Research & Therapy Online Firstpub Date.

[3] Wu Y, Beiser AS, Breteler MMB, Fratiglioni L, Helmer C, Hendrie HC, Honda H, Ikram MA, Langa KM, Lobo A, Matthews FE, Ohara T, Pérès K, Qiu C, Seshadri S, Sjölund B, Skoog I, Brayne C (2017). The changing prevalence and incidence of dementia over time-current evidence. Nat Rev Neurol.

[4] Knapp M, Prince M, Albanese E, Banerjee S, Dhanasiri S, & Fernandez JL (2007). https://caregiversprommd-project.eu/wp-content/uploads/Dementia_UK_Full_Report-1.pdf.

[5] Prince M, Knapp M, Guerchet M, McCrone P, Prina M, Comas-Herrera A, Wittenberg R, Adelaja B, Hu B, King D, Rehill A, Salimkumar D (2014). https://www.alzheimers.org.uk/sites/default/files/migrate/downloads/dementia_uk_update.pdf.

[6] Miranda-Castillo C, Woods B, Orrell M (2010). People with dementia living alone: what are their needs and what kind of support are they receiving?. Int Psychogeriatr.

[7] (2015). Dementia Care.

[8] Lewis F, Karlsberg Schaffer S, Sussex J, O'Neill P, Cockcroft L (2014). https://www.ohe.org/publications/trajectory-dementia-uk-making-difference.

[9] Wimo A, Jönsson Linus, Bond J, Prince M, Winblad B, Alzheimer Disease International (2013). The worldwide economic impact of dementia 2010. Alzheimers Dement.

[10] (2011). Department of Health.

[11] Boaden Andrew (2016). Alzheimer’s Society.

[12] Dominic C (2016). Alzheimer’s Society.

[13] All-Party Parliamentary Group on Dementia (2016). Alzheimer’s Society.

[14] McKechnie V, Barker C, Stott J (2014). The effectiveness of an Internet support forum for carers of people with dementia: a pre-post cohort study. J Med Internet Res.

[15] Meiland F, Innes A, Mountain G, Robinson L, van der Roest H, García-Casal J Antonio, Gove D, Thyrian R, Evans S, Dröes Rose-Marie, Kelly F, Kurz A, Casey D, Szcześniak Dorota, Dening T, Craven P, Span M, Felzmann H, Tsolaki M, Franco-Martin M (2017). Technologies to Support Community-Dwelling Persons With Dementia: A Position Paper on Issues Regarding Development, Usability, Effectiveness and Cost-Effectiveness, Deployment, and Ethics. JMIR Rehabil Assist Technol.

[16] Samsi K, Abley C, Campbell S, Keady J, Manthorpe J, Robinson L, Watts S, Bond J (2014). Negotiating a labyrinth: experiences of assessment and diagnostic journey in cognitive impairment and dementia. Int J Geriatr Psychiatry.

[17] Campbell S, Manthorpe J, Samsi K, Abley C, Robinson L, Watts S, Bond J, Keady J (2016). Living with uncertainty: Mapping the transition from pre-diagnosis to a diagnosis of dementia. J Aging Stud.

[18] Moon H, Adams KB (2013). The effectiveness of dyadic interventions for people with dementia and their caregivers. Dementia (London).

[19] Merrick K, Camic P M, O'Shaughnessy M (2013). Couples constructing their experiences of dementia: A relational perspective. Dementia.

[20] McGovern J (2011). Couple meaning-making and dementia: challenges to the deficit model. J Gerontol Soc Work.

[21] MacRae H (2011). Self and other: The importance of social interaction and social relationships in shaping the experience of early-stage Alzheimer's disease. Journal of Aging Studies.

[22] Hellström I, Nolan M, Lundh U (2007). Sustaining couplehood 'Spouses' strategies for living positively with dementia. Dementia.

[23] Bielsten T, Hellström Ingrid (2017). A review of couple-centred interventions in dementia: Exploring the what and why - Part A. Dementia.

[24] Bielsten T, Hellström Ingrid (2017). An extended review of couple-centred interventions in dementia: Exploring the what and why - Part B. Dementia.

[25] (2016). SOU.

[26] (2012). Department of Health.

[27] Keady J, Campbell S, Barnes H, Ward R, Li X, Swarbrick C, Burrow S, Elvish R (2012). Neighbourhoods and dementia in the health and social care context: a realist review of the literature and implications for UK policy development. Reviews in Clinical Gerontology.

[28] Klimova B (2017). Mobile Phone Apps in the Management and Assessment of Mild Cognitive Impairment and/or Mild-to-Moderate Dementia: An Opinion Article on Recent Findings. Front Hum Neurosci.

[29] Bateman D R, Srinivas B, Emmett T W, Schleyer T K, Holden R J, Hendrie H C, Callahan C M (2017). Categorizing Health Outcomes and Efficacy of mHealth Apps for Persons With Cognitive Impairment: A Systematic Review. J Med Internet Res.

[30] Faucounau V, Riguet M, Orvoen G, Lacombe A, Rialle V, Extra J, Rigaud A-s (2009). Electronic tracking system and wandering in Alzheimer's disease: a case study. Ann Phys Rehabil Med.

[31] Pot A M, Willemse B M, Horjus S (2012). A pilot study on the use of tracking technology: feasibility, acceptability, and benefits for people in early stages of dementia and their informal caregivers. Aging Ment Health.

[32] Thorpe J R, Rønn-Andersen K V H, Bień P, Özkil A G, Forchhammer B H, Maier A M (2016). Pervasive assistive technology for people with dementia: a UCD case. Healthcare Technology Letters.

[33] Christina Y, Jean F C, Marc K, Shannon J (2013). Mobile app development and usability research to help dementia and Alzheimer patients. 2013 IEEE Long Island Systems, Applications and Technology Conference (LISAT).

[34] Lancaster GA, Dodd S, Williamson PR (2004). Design and analysis of pilot studies: recommendations for good practice. J Eval Clin Pract.

[35] Bowen D, Kreuter M, Spring B, Cofta-Woerpel L, Linnan L, Weiner D, Bakken S, Kaplan C, Squiers L, Fabrizio C, Fernandez M (2009). How we design feasibility studies. American Journal of Preventive Medicine.

[36] Craig P, Dieppe P, Macintyre S, Michie S, Nazareth I, Petticrew M, Medical Research Council Guidance (2008). Developing and evaluating complex interventions: the new Medical Research Council guidance. BMJ.

[37] Judge KS, Yarry SJ, Orsulic-Jeras S (2010). Acceptability and feasibility results of a strength-based skills training program for dementia caregiving dyads. Gerontologist.

[38] Brooke J, Jordan P W, Thomas B, McClelland I L, Weerdmeester B (1996). SUS-A quick and dirty usability scale. Usability evaluation in industry.

[39] Logsdon R, Gibbons L, McCurry S, Teri L (1999). Quality of life in Alzheimer's disease: patient and caregiver reports. Journal of Mental Health and Aging.

[40] Brouwer W, van Exel N, van Gorp B, Redekop W (2006). The CarerQol instrument: a new instrument to measure care-related quality of life of informal caregivers for use in economic evaluations. Quality of Life Research.

[41] Archbold PG, Stewart BJ, Greenlick MR, Harvath T (1990). Mutuality and preparedness as predictors of caregiver role strain. Res Nurs Health.

[42] Aron A, Aron E, Smollan D (1992). Inclusion of Other in the Self Scale and the structure of interpersonal closeness. Journal of personality and social psychology.

[43] Schwarzer R, Jerusalem M (1995). Generalized Self-Efficacy scale. Measures in Health Psychology: A User's Portfolio.

[44] Herdman M, Gudex C, Lloyd A, Janssen M, Kind P, Parkin D, Bonsel G, Badia X (2011). Development and preliminary testing of the new five-level version of EQ-5D (EQ-5D-5L). Qual Life Res.

[45] Smith SC, Lamping DL, Banerjee S, Harwood R, Foley B, Smith P, Cook JC, Murray J, Prince M, Levin E, Mann A, Knapp M (2005). Measurement of health-related quality of life for people with dementia: development of a new instrument (DEMQOL) and an evaluation of current methodology. Health Technol Assess.

[46] Orsmond GI, Cohn ES (2015). The Distinctive Features of a Feasibility Study: Objectives and Guiding Questions. OTJR (Thorofare N J).

[47] Hellström I, Nolan M, Nordenfelt L, Lundh U (2007). Ethical and methodological issues in interviewing persons with dementia. Nurs Ethics.

[48] Dewing J (2008). Process consent and research with older persons living with dementia. Research Ethics Review.

[49] (2018). Health Research Authority.

[50] (2015). O'Shaughnessy A.

