# Supplementary material for: Designing and Implementing a Home-Based Couple Management Guide for Couples Where One Partner has Dementia (DemPower): Protocol for a Nonrandomized Feasibility Trial
Source: JMIR Res Protoc. 2018 Aug 10;7(8):e171. doi: 10.2196/resprot.9087 (PMC6109228; doi:10.2196/resprot.9087)
Supplement: Multimedia Appendix 1 [file resprot_v7i8e171_app1.pdf]

Process Evaluation Questionnaire

Participant ID number:______________

(To be administered by the researcher at post intervention point)

The following questions refer to various aspects of the study process. Your responses will help us find out what went well and what didn't and how we could improve the process. Please answer all questions.

1. Where did you hear about the research? Please tick a relevant option listed below.

Someone from hospital phoned us (CRN) ☐
Researcher contacted us (JDR) ☐
Through a group/dementia café ☐
Saw a poster at the memory clinic ☐
I/family/friend saw an advert online ☐

2. Was the information sheet clear and easy to understand?

☐ Yes ☐ No

3. Was the invitation letter clear?

☐ Yes ☐ No

4. Was the consent form clear?

☐ Yes ☐ No

5. Did the researcher explain the study well before you made your decision about your participation?

☐ Yes ☐ No

6. Would you still consider participating in the study, if you were told that there would be two groups in the study where one group will receive the app and other group will receive a paper copy of dementia services and you couldn't choose which group you would be in.

☐ ☐

Yes No

7. Baseline assessment – Secondary outcome

a) Were the assessments/questionnaires tolerable?

☐ Yes ☐ No

b) Did the assessments take too long?

☐ Yes ☐ No

c) Did you find it hard to respond to any questions in the assessments? If yes, explain

☐ Yes ☐ No

If yes, explain

____
____
____

d) Did the researcher explain the assessment and the purpose?

☐ Yes ☐ No

e) Did you understand what was happening?

☐ Yes ☐ No

8. Home visits & telephone calls

a) How many times did the researcher visit you?

____
____

b) What was the purpose of visit? Please tick from the following

Discuss the study ☐

Sign the consent form ☐

To hand in the device ☐

Provide a tutorial on using the device & the app ☐

To provide technical support ☐

Other ☐

If other, please specify

____

____

c) How many times did the researcher phone you?

____

____

____

d) Did you find that the phone calls were more frequent than required?

☐ Yes ☐ No

e) Did you find the phone calls were less frequent than required?

☐ Yes ☐ No

f) Did you understand the purpose of the phone calls?

☐ Yes ☐ No

If no, please explain:

____

____

g) How many times did you phone the researcher?

h) What were the purposes of your calls?

i) Did the phone calls take place at a time convenient for you?

☐ Yes ☐ No

j) Did the home visits take place at a time convenient for you?

☐ Yes ☐ No

If no, please explain:

k) What did you think about the length of home visits?

9. Did the researcher provide you enough guidance on how to use the device and app?

☐ Yes ☐ No

If no, please explain:

10. Did you see any value in the activities you performed as a part of DemPower?

☐ Yes ☐ No

Please explain:

11. Did you find it useful to write your reflections down?

☐ Yes ☐ No

Please explain:

____
____
____

12. Did you find discussing your reflections with your partner/spouse useful?

☐ Yes ☐ No

Please explain:

____
____
____

13. Was the introduction video clear enough?

☐ Yes ☐ No

Could anything be improved:

____
____
____

14. Did you know what was expected of you during the study?

☐ Yes ☐ No

Please explain:

____
____
____

15. Did the introduction clarify how to engage with the guide?

☐ Yes ☐ No

Please explain:

____
____
____

16. Did you like DemPower?

☐ Yes ☐ No

17. What worked for you?

____
____
____
____

18. What didn't you like?

____
____
____

19. What would you like to be done differently?

____
____
____

20. Were there any activities that you disliked, or found too difficult?

☐ Yes ☐ No

If yes please explain

____
____
____

21. Did you find it easy to engage with DemPower?

☐ Yes ☐ No

____
____
____

22. Did you feel that DemPower fit with your lifestyle?

☐ Yes ☐ No

Please explain:

23. When and how did you find time to engage with DemPower together?

24. Do you plan to continue any of the activities suggested in the DemPower guide?

☐ Yes ☐ No

a) Which activities do you think you will continue to do?

b) **If not, can you please tell us why you think you will not continue?**

c) Do you feel DempPower is something that you could carry on doing in the long term ?

☐ Yes ☐ No

d) Do you feel DempPower is something that you could return to anytime?

☐ Yes ☐ No

25. Instructions

a) Did the help videos explain clearly all the instructions?

☐ Yes ☐ No

b) Did the text instructions convey the message clearly?

☐ Yes ☐ No

c) Were there any parts of the instructions that were unclear?

☐ Yes ☐ No

Please specify:

____

____

____

d) Have you got any suggestions for any parts of the instructions that could be improved?

____

____

____

e) Is there any additional information you can think of which would be useful?

____

____

____

f) Which help did you use the most? Please mark one of the following.

Paper help ☐

Video help ☐

Please explain:

____

____

____

26. Using a Tablet

a) **Was the screen size acceptable for the amount of reading and watching you had to do?**

☐ Yes ☐ No

Please explain:

b) **Were you able to increase/decrease the text size easily?**

☐ Yes ☐ No

c) **Were you able to switch text to speech function easily?**

☐ Yes ☐ No

d) **How comfortable were you with using a touchscreen device?**

☐ A great deal ☐ Somewhat ☐ Not at all

e) **Were you comfortable navigating your way through DemPower?**

☐ A great deal ☐ Somewhat ☐ Not at all

f) **Were the navigational instructions clear enough?**

☐ Yes ☐ No

g) **Did you feel that you had enough support to be able to use the tablet?**

☐ Yes ☐ No

h) **Did you feel that family and friends were supportive of you using the tablet?**

☐ Yes ☐ No

i) Did their support influence how you used it?

☐ Yes ☐ No

Please explain:

____
____
____

j) Did the ‘application’ work reliably?

☐ Yes ☐ No

If no, please explain:

____
____
____

k) Did you have any issues with the battery life?

☐ Yes ☐ No

27. Have you got any further comments?

____
____
____
