# Supplementary material for: Designing and Implementing a Home-Based Couple Management Guide for Couples Where One Partner has Dementia (DemPower): Protocol for a Nonrandomized Feasibility Trial
Source: JMIR Res Protoc. 2018 Aug 10;7(8):e171. doi: 10.2196/resprot.9087 (PMC6109228; doi:10.2196/resprot.9087)
Supplement: Multimedia Appendix 2 [file resprot_v7i8e171_app2.pdf]

Questionnaire for evaluating the feasibility and acceptability of DemPower

Participant ID number:_____________

The following questions refer to **'Home and Neighbourhood'** part of the DemPower guide. Your responses will help us find out what you think of the guide and give us feedback on its contents and activities. Please answer all questions.

1. **Please rate how well the theme 'Home and Neighbourhood' addressed aspects of your daily life**

☐ A great deal	☐ Somewhat	☐ Not at all

If your answer is somewhat or Not at all, please tell us why you have chosen this answer

____
____
____
____
____
____
____
____
____
____

2. **Please tick all the sections that you have completed**

The meaning of home	☐ yes	☐ No
Inside your home	☐ yes	☐ No
The outdoors	☐ yes	☐ No
Couplehood	☐ yes	☐ No

If you have answered no to any of the above, please tell us why

____

3. Did you find these sections helpful?

The meaning of home	☐ A great deal	☐ Somewhat	☐ Not at all	☐ Not applicable
Inside your home	☐ A great deal	☐ Somewhat	☐ Not at all	☐ Not applicable
The outdoors	☐ A great deal	☐ Somewhat	☐ Not at all	☐ Not applicable
Couplehood	☐ A great deal	☐ Somewhat	☐ Not at all	☐ Not applicable

Could anything be improved? Please specify:

____

4. Were the videos easy to understand?

☐ Easy ☐ Not so easy ☐ Difficult

5. Do you think the guide has helped you to think and talk about

**Your life together at home**	☐ A great deal	☐ Somewhat	☐ Not at all	☐ Not applicable
**The meaning of home to you both**	☐ A great deal	☐ Somewhat	☐ Not at all	☐ Not applicable
**What is good about your current home**	☐ A great deal	☐ Somewhat	☐ Not at all	☐ Not applicable
**How to adapt your home**	☐ A great deal	☐ Somewhat	☐ Not at all	☐ Not applicable
**The importance of spending time outdoors**	☐ A great deal	☐ Somewhat	☐ Not at all	☐ Not applicable
**Consider using support devices (personal locators, easy to use phone, ID card etc)**	☐ A great deal	☐ Somewhat	☐ Not at all	☐ Not applicable
**Know whom to contact to discuss your support needs**	☐ A great deal	☐ Somewhat	☐ Not at all	☐ Not applicable
**Your relationship with each other**	☐ A great deal	☐ Somewhat	☐ Not at all	☐ Not applicable
**Your expectations of each other**	☐ A great deal	☐ Somewhat	☐ Not at all	☐ Not applicable

6. **Do you feel that the guide has helped you to focus on your strengths and the things that you can do?**

☐ A great deal ☐ Somewhat ☐ Not at all

7. **Do you consider the activity of taking photos of important things and places as a positive experience**

☐ A great deal ☐ Somewhat ☐ Not at all

8. **Do you consider your discussion about adapting the home as important.**

☐ A great deal ☐ Somewhat ☐ Not at all

9. **Do you consider the activity of taking a walk in the neighbourhood and taking pictures of your favourite places as a positive experience**

☐ A great deal ☐ Somewhat ☐ Not at all

10. We consider the discussion about experiences that brought us closer as meaningful

☐ A great deal ☐ Somewhat ☐ Not at all

11. We consider the selection of a song and listening to it together as a positive experience

☐ A great deal ☐ Somewhat ☐ Not at all

12. Did you find the information about adapting our home important?

☐ A great deal ☐ Somewhat ☐ Not at all

13. Did you find the information about support items (assistive technology) important?

☐ A great deal ☐ Somewhat ☐ Not at all

14. Please tell us how helpful the inbuilt help video has been

☐ A great deal ☐ Somewhat ☐ Not at all

Could anything be improved? Please specify:

____
____
____
____
____
____
____
____

15. Please tell us how easy or difficult it was to make time for the guide in your weekly schedule?

☐ Easy   ☐ Not so easy   ☐ Difficult
