# Supplementary material for: Designing and Implementing a Home-Based Couple Management Guide for Couples Where One Partner has Dementia (DemPower): Protocol for a Nonrandomized Feasibility Trial
Source: JMIR Res Protoc. 2018 Aug 10;7(8):e171. doi: 10.2196/resprot.9087 (PMC6109228; doi:10.2196/resprot.9087)
Supplement: Multimedia Appendix 3 [file resprot_v7i8e171_app3.pdf]

Questionnaire for evaluating the feasibility and acceptability of DemPower

Participant ID number:_____________

The following questions refer to '**Meaningful activities and relationships**' part of the DemPower guide. Your responses will help us find out what you think of the guide and give us feedback on its contents and activities. Please answer all questions.

1. Please rate how well the theme 'Meaningful activities and relationships' addressed aspects of your daily life

☐ A great deal	☐ Somewhat	☐ Not at all

If your answer is somewhat or Not at all, please tell us why you have chosen this answer

____
____
____
____
____
____
____
____
____
____

2. Please tick all the sections that you have completed

Physical exercise	☐ Yes	☐ No
Doing things together at home and outside	☐ Yes	☐ No
Individual activities	☐ Yes	☐ No
Adapting activity to capability	☐ Yes	☐ No
Mental Exercise	☐ Yes	☐ No

If you have answered no to any of the above, please tell us why

3. Did you find these sections helpful?

Physical exercise	☐ A great deal	☐ Somewhat	☐ Not at all
Doing things together at home and outside	☐ A great deal	☐ Somewhat	☐ Not at all
Individual activities	☐ A great deal	☐ Somewhat	☐ Not at all
Adapting activity to capability	☐ A great deal	☐ Somewhat	☐ Not at all
Mental Exercise	☐ A great deal	☐ Somewhat	☐ Not at all

Could anything be improved? Please specify:

4. Were the videos easy to understand?

☐ Easy ☐ Not so easy ☐ Difficult

5. **Did the video on physical exercise present clear instructions on how to do these exercises on your own?**

☐ A great deal	☐ Somewhat	☐ Not at all

Could anything be improved? Please specify:

____
____
____
____
____
____
____
____
____
____

6. **Did the guide help you to think and identify different ways you could build your physical fitness?**

☐ A great deal	☐ Somewhat	☐ Not at all

7. **Do you consider the physical exercise as a positive experience**

☐ A great deal	☐ Somewhat	☐ Not at all

8. **Please tell us how helpful were the suggestions for involving each other in daily chores, such as shopping, cleaning, gardening etc.**

☐ A great deal	☐ Somewhat	☐ Not at all

If you didn't find these helpful or meaningful, please explain:

____

9. Do you consider the list you made of activities/chores to do together as useful?

☐ A great deal ☐ Somewhat ☐ Not at all

10. Please tell us how helpful was the information on individual activities?

☐ A great deal ☐ Somewhat ☐ Not at all

Could anything be improved? Please specify:

____

11. Were you both able to think of some activities that you enjoy individually and to do on your own?

☐ A great deal ☐ Somewhat ☐ Not at all

Please explain if you haven't been able to do this:

____

____
____
____
____
____

12. Do you consider the discussion about and schedule of individual activities as important?

A great deal	Somewhat	Not at all

13. Did the videos on adapting activity to capability hep you focus on your abilities?

A great deal	Somewhat	Not at all

14. Did the discussion about 'adapting activity to capability' help you think of how you can keep doing activities that are important to you?

A great deal	Somewhat	Not at all

15. Do you consider the suggestions for mental activity as useful?

A great deal	Somewhat	Not at all

16. Did the selected games and activities provide you with a positive experiences

A great deal	Somewhat	Not at all

Please explain:

____
____
____
____
____
____
____
____
____
____

17. Did the guide help you to think and identify different ways you could improve your mental abilities?

☐	☐	☐
A great deal	Somewhat	Not at all

18. Please tell us if the information and activities In this part help you spend meaningful time together

☐	☐	☐
A great deal	Somewhat	Not at all

19. Did you find the information about physical exercise important and useful?

☐	☐	☐
A great deal	Somewhat	Not at all

20. Did you find the information about mental exercise important and useful?

☐	☐	☐
A great deal	Somewhat	Not at all

21. Please tell us how helpful the inbuilt help video has been

☐	☐	☐
A great deal	Somewhat	Not at all

Could anything be improved? Please specify:

____
____
____
____
____
____
____
____

22. Please tell us how easy or difficult it was to make time for the guide in your weekly schedule?

☐	☐	☐
Easy	Not so easy	Difficult
