# Supplementary material for: Designing and Implementing a Home-Based Couple Management Guide for Couples Where One Partner has Dementia (DemPower): Protocol for a Nonrandomized Feasibility Trial
Source: JMIR Res Protoc. 2018 Aug 10;7(8):e171. doi: 10.2196/resprot.9087 (PMC6109228; doi:10.2196/resprot.9087)
Supplement: Multimedia Appendix 4 [file resprot_v7i8e171_app4.pdf]

# Questionnaire for evaluating the feasibility and acceptability of DemPower

**Participant ID number:_____________**

The following questions refer to '**Meeting, sharing and caring in your neighbourhood**' part of the DemPower guide. Your responses will help us find out what you think of the guide and give us feedback on its contents and activities. Please answer all questions.

1. **Please rate how well the theme 'Meeting, sharing and caring in your neighbourhood' addressed aspects of your daily life**

| ☐ | ☐ | ☐ |
|---|---|---|
| A great deal | Somewhat | Not at all |

**If your answer is somewhat or Not at all, please tell us why you have chosen this answer**

______________________________________________________________________________
______________________________________________________________________________
______________________________________________________________________________
______________________________________________________________________________
______________________________________________________________________________
______________________________________________________________________________
______________________________________________________________________________
______________________________________________________________________________
______________________________________________________________________________
__________

2. **Please tick all the sections that you have completed**

| | | |
|---|---|---|
| Socialising with family and friends | ☐ Yes | ☐ No |
| Meeting others who live with dementia | ☐ Yes | ☐ No |
| Informing Others | ☐ Yes | ☐ No |

**If you have answered no to any of the above, please tell us why**

______________________________________________________________________________
______________________________________________________________________________

______________________________________________________________________________
______________________________________________________________________________
______________________________________________________________________________
______________________________________________________________________________
______________________________________________________________________________
______________________________________________________________________________
______________________________________________________________________________
_________

**3. Did you find these sections helpful?**

| | | | |
|---|---|---|---|
| Socialising with family and friends | ☐ A great deal | ☐ Somewhat | ☐ Not at all |
| Meeting others who live with dementia | ☐ A great deal | ☐ Somewhat | ☐ Not at all |
| Informing Others | ☐ A great deal | ☐ Somewhat | ☐ Not at all |

Could anything be improved? Please specify:

______________________________________________________________________________
______________________________________________________________________________
______________________________________________________________________________
______________________________________________________________________________
______________________________________________________________________________
______________________________________________________________________________
______________________________________________________________________________
______________________________________________________________________________
______________________________________________________________________________
_________

**4. Were the videos easy to understand?**

☐ Easy ☐ Not so easy ☐ Difficult

**5. Do you think that planning a schedule to meet with friends and family and putting it in your monthly diary helpful?**

☐ A great deal ☐ Somewhat ☐ Not at all

Please explain:

______________________________________________________________________
______________________________________________________________________
______________________________________________________________________
______________________________________________________________________
______________________________________________________________________
______________________________________________________________________
______________________________________________________________________
______________________________________________________________________
______________________________________________________________________
________

**6. Did the guide encourage you to meet people whom you know**

| | | |
|---|---|---|
| ☐ | ☐ | ☐ |
| A great deal | Somewhat | Not at all |

**7. Did you feel happy and positive about meeting people whom you know**

| | | |
|---|---|---|
| ☐ | ☐ | ☐ |
| A great deal | Somewhat | Not at all |

Please explain:

______________________________________________________________________
______________________________________________________________________
______________________________________________________________________
______________________________________________________________________
______________________________________________________________________

**8. Did the guide encourage you to meet people whom you don't know**

| | | |
|---|---|---|
| ☐ | ☐ | ☐ |
| A great deal | Somewhat | Not at all |

**9. Did you feel happy and positive about meeting people whom you don't know**

| | | |
|---|---|---|
| ☐ | ☐ | ☐ |
| A great | Somewhat | Not at all |

deal

Please explain:

______________________________________________________________________________
______________________________________________________________________________
______________________________________________________________________________
______________________________________________________________________________
______________________________________________________________________________

**10. Please tell us how helpful was the information about groups for people with dementia and their carers?**

| | | |
|---|---|---|
| ☐ | ☐ | ☐ |
| A great deal | Somewhat | Not at all |

**11. Did you consider attending any groups?**

| | |
|---|---|
| ☐ | ☐ |
| Yes | No |

Please explain:

______________________________________________________________________________
______________________________________________________________________________
______________________________________________________________________________
______________________________________________________________________________
______________________________________________________________________________

**12. Did you consider meeting others who live with dementia as important?**

| | | |
|---|---|---|
| ☐ | ☐ | ☐ |
| A great deal | Somewhat | Not at all |

If not, please explain:

______________________________________________________________________________
______________________________________________________________________________
______________________________________________________________________________
______________________________________________________________________________
______________________________________________________________________________

**13. Did you share your experience of dementia and how it affects your life with your neighbours or others**

☐ Yes ☐ No

**14. Do you think it helped others understand your life a little better**

☐ A great deal ☐ Somewhat ☐ Not at all

**15. Did you find it helpful to share your experience**

☐ A great deal ☐ Somewhat ☐ Not at all

**16. Did you find sharing your experience hurtful and burdensome**

☐ A great deal ☐ Somewhat ☐ Not at all

Please explain:

______________________________________________________________
______________________________________________________________
______________________________________________________________
______________________________________________________________
______________________________________________________________

**17. Do you think that the guide has helped you to think and talk about**

| | | | |
|---|---|---|---|
| **Socialising with family** | ☐ A great deal | ☐ Somewhat | ☐ Not at all |
| **Socialising with friends** | ☐ A great deal | ☐ Somewhat | ☐ Not at all |
| **Give you ideas for socialising** | ☐ A great deal | ☐ Somewhat | ☐ Not at all |
| **Your experiences of these meetings** | ☐ A great deal | ☐ Somewhat | ☐ Not at all |

| | | | |
|---|---|---|---|
| **Importance of sharing your experience with others** | ☐ A great deal | ☐ Somewhat | ☐ Not at all |

**18. Did you share the fact sheet 'How to engage' with your family and friends?**

☐ Yes ☐ No

If no, please explain:

______________________________________________________________
______________________________________________________________
______________________________________________________________
______________________________________________________________
______________________________________________________________

**19. Please tell us if the fact sheet is helpful**

☐ A great deal ☐ Somewhat ☐ Not at all

**20. Please tell us how helpful the inbuilt help video has been?**

☐ A great deal ☐ Somewhat ☐ Not at all

Could anything be improved? Please specify:

______________________________________________________________
______________________________________________________________
______________________________________________________________
______________________________________________________________
______________________________________________________________
______________________________________________________________
______________________________________________________________
______________________________________________________________
______________________________________________________________
________

**21. Please tell us how easy or difficult it was to make time for the guide in your weekly schedule?**

☐ Easy ☐ Not so easy ☐ Difficult

If difficult, please explain:

___________________________________________

___________________________________________

___________________________________________

___________________________________________

___________________________________________

___________________________________________
