# Supplementary material for: Designing and Implementing a Home-Based Couple Management Guide for Couples Where One Partner has Dementia (DemPower): Protocol for a Nonrandomized Feasibility Trial
Source: JMIR Res Protoc. 2018 Aug 10;7(8):e171. doi: 10.2196/resprot.9087 (PMC6109228; doi:10.2196/resprot.9087)
Supplement: Multimedia Appendix 5 [file resprot_v7i8e171_app5.pdf]

Questionnaire for evaluating the feasibility and acceptability of DemPower

Participant ID number:______________

The following questions refer to '**Managing emotions, communication and approach**' part of the DemPower guide. Your responses will help us find out what you think of the guide and give us feedback on its contents and activities. Please answer all questions.

1. **Please rate how well the theme 'Managing emotions, communication and approach' addressed aspects of your daily life**

☐ A great deal	☐ Somewhat	☐ Not at all

If your answer is somewhat or Not at all, please tell us why you have chosen this answer

____
____
____
____
____
____
____
____
____
____

2. **Please tick all the sections that you have completed**

Being a comfort and a friend	☐ Yes	☐ No
Living as usual and keeping the routine	☐ Yes	☐ No
Stress	☐ Yes	☐ No
Conflicts	☐ Yes	☐ No
Future and planning	☐ Yes	☐ No
Communication	☐ Yes	☐ No

If you have answered no to any of the above, please tell us why

____
____
____
____
____
____
____
____
____
____

3. Did you find these sections helpful?

Being a comfort and a friend	☐ A great deal	☐ Somewhat	☐ Not at all
Living as usual and keeping the routine	☐ A great deal	☐ Somewhat	☐ Not at all
Stress	☐ A great deal	☐ Somewhat	☐ Not at all
Conflicts	☐ A great deal	☐ Somewhat	☐ Not at all
Future and planning	☐ A great deal	☐ Somewhat	☐ Not at all
Communication	☐ A great deal	☐ Somewhat	☐ Not at all

Could anything be improved? Please specify:

____
____
____
____
____
____
____
____

4. Were the videos easy to understand?

☐ Easy ☐ Not so easy ☐ Difficult

5. Do you think that the guide has helped you to think and talk about

**Different roles you take in your relationship**	☐ A great deal	☐ Somewhat	☐ Not at all
**The need to be a friend**	☐ A great deal	☐ Somewhat	☐ Not at all
**Continuing to live as usual**	☐ A great deal	☐ Somewhat	☐ Not at all
**The importance of keeping a routine**	☐ A great deal	☐ Somewhat	☐ Not at all
**Situations that stress you**	☐ A great deal	☐ Somewhat	☐ Not at all
**Different way you could relax**	☐ A great deal	☐ Somewhat	☐ Not at all
**How to deal with conflict situations**	☐ A great deal	☐ Somewhat	☐ Not at all
**Financial/legal matters**	☐ A great deal	☐ Somewhat	☐ Not at all
**Care needs**	☐ A great deal	☐ Somewhat	☐ Not at all
**Living arrangements**	☐ A great deal	☐ Somewhat	☐ Not at all
**Health care decision maker**	☐ A great deal	☐ Somewhat	☐ Not at all
**Emergency contact**	☐ A great deal	☐ Somewhat	☐ Not at all

6. Did you discuss the methods you use to comfort each other

☐ A great deal	☐ Somewhat	☐ Not at all

7. Did you speak about the support you need from each other?

☐ A great deal	☐ Somewhat	☐ Not at all

8. Did you find the contact information for counselling services/ other organisations helpful?

☐ A great deal	☐ Somewhat	☐ Not at all

9. How helpful were the videos on living as usual

☐ A great deal	☐ Somewhat	☐ Not at all

Please explain:

____
____
____
____
____
____

10. Do you have a planned routine?

☐ Yes	☐ No

11. Did you find the discussion around planning a routine helpful?

☐ A great deal	☐ Somewhat	☐ Not at all

12. Please tell us if having a routine helped improve sense of familiarity, comfort and

control

**For you with dementia**	☐ A great deal	☐ Somewhat	☐ Not at all
**For you as a carer spouse/partner**	☐ A great deal	☐ Somewhat	☐ Not at all

13. Please tell us how helpful were the videos on managing stress?

☐ A great deal ☐ Somewhat ☐ Not at all

If you didn't find these helpful, Please explain

____
____
____
____
____

14. Did you find the information on whom to contact for support if you are unable to cope with stress helpful?

☐ A great deal ☐ Somewhat ☐ Not at all

If not, please explain

____
____
____
____
____

15. Did you make a list of methods/ approaches that has helped you both to deal with conflicts in your relationship?

☐ Yes ☐ No

a) Please tell us if you found this exercise helpful?

☐ A great deal	☐ Somewhat	☐ Not at all

16. Please tell us how helpful was the section on ‘future and planning’?

☐ A great deal	☐ Somewhat	☐ Not at all

Please explain:

____
____
____
____
____
____
____
____
____
____

17. Did the guide provide useful communication tips?

☐ A great deal	☐ Somewhat	☐ Not at all

18. Did you think of and talk to each other about the methods you use in everyday communication?

☐ Yes	☐ No

19. Did you find the discussion about your own strategies for communication helpful?

☐ A great deal	☐ Somewhat	☐ Not at all

Please explain:

____
____
____
____
____

20. Please tell us how easy or difficult it was to make time for the guide in your weekly schedule?

☐ Easy ☐ Not so easy ☐ Difficult

If difficult, please explain:

____
____
____
____
____
____

21. Please tell us how useful was the inbuilt help video?

☐ A great deal	☐ Somewhat	☐ Not at all

Could anything be improved? Please specify:

____
____
____
____
____
____
____
____
____
____
